# Trends and Challenges in Orthopedic Referrals Following the Development of a General Medicine Department in a Community Hospital: A Mixed-Methods Study

**DOI:** 10.7759/cureus.103382

**Published:** 2026-02-10

**Authors:** Kasumi Nishikawa, Ryuichi Ohta, Chiaki Sano

**Affiliations:** 1 Community Care, Unnan City Hospital, Unnan, JPN; 2 Community Medicine Management, Shimane University Faculty of Medicine, Izumo, JPN

**Keywords:** community, family medicine, general medicine, general practice, hospitals, interprofessional relations, orthopedic procedures, patient care team, qualitative research, rural

## Abstract

Background

In community hospitals, an increasing number of older patients with multimorbidity are being admitted to orthopedic wards. Although general medicine departments are expected to contribute to comprehensive inpatient management, their roles in orthopedic ward care and interdepartmental collaboration remain insufficiently defined. This study aimed to explore how the establishment of a general medicine department influenced collaboration with orthopedics, and to identify challenges and opportunities for improving inpatient care in a community hospital setting.

Methods

We conducted an explanatory sequential mixed-methods study at a community hospital in rural Japan. First, a retrospective review of electronic medical records was performed to analyze referral patterns from orthopedics to general medicine between April 2016 and March 2024. Descriptive statistics were used to examine patient characteristics, referral timing, and reasons for consultation. Subsequently, semi-structured interviews were conducted with 11 general medicine physicians, and qualitative data were analyzed using thematic analysis to explore perceptions of collaboration, roles, and system-level challenges.

Results

A total of 530 orthopedic inpatients were referred to general medicine during the study period, with consistently high mean patient age. Referrals varied widely in timing and content, with symptoms, laboratory abnormalities, and vital sign abnormalities being the most common reasons. Qualitative analysis identified four themes: (i) awkwardness in the referral process arising from differences in professional values; (ii) building bridges toward effective collaboration; (iii) mutual collaboration grounded in an appropriate sense of responsibility; and (iv) the importance of mutual understanding for sustainable collaboration. Participants emphasized the benefits of early general medicine involvement for comprehensive management and patient safety, while highlighting challenges related to role ambiguity, workload imbalance, and lack of standardized referral criteria.

Conclusions

In community hospitals, general medicine departments can play a critical role in improving comprehensive care and safety for orthopedic inpatients. However, sustainable collaboration requires clearer role definition, standardized referral processes, and organizational support. Redefining general medicine as a core component of inpatient care may enhance interdepartmental collaboration and improve overall quality of care in community hospital settings.

## Introduction

In community hospitals, general medicine departments play a central role in providing comprehensive care for hospitalized patients with multimorbidity, including reassessing diagnoses and integrating management across multiple conditions [1]. With the rapid aging of the population, the number of patients with various coexisting chronic diseases has increased substantially, and the involvement of general medicine has been shown to facilitate smoother clinical care and more effective management of complications [2,3]. In addition, general medicine departments often coordinate inpatient care processes, from clinical management after admission to discharge planning, thereby promoting earlier discharge and more efficient use of hospital beds [4]. Owing to these functions, general medicine serves as a key hub for interdepartmental collaboration within hospitals. It plays an essential role in maintaining the quality of care for patients referred from other specialties.

In community hospital settings, the ability of general medicine departments to respond flexibly and effectively to referrals from specialty departments is closely linked to the overall quality of inpatient care [5]. Referrals from orthopedic departments, in particular, frequently involve patients with fractures or joint disorders who also have coexisting chronic conditions such as hypertension, diabetes mellitus, and cardiovascular disease. In this context, comprehensive multimorbidity management by general medicine may improve the overall quality of inpatient care by optimizing perioperative risk management and stabilizing patients’ general medical conditions [6]. Moreover, because the content and level of detail in referral information strongly influence clinical decision-making in general medicine, effective information sharing and collaboration between departments are essential [7].

Despite the importance of interdepartmental collaboration, there remains a lack of research evaluating the nature of collaboration between general medicine and other specialties in community hospitals, particularly regarding temporal changes in the quality and volume of referral content. Specifically, there is limited empirical evidence on the clinical backgrounds underlying referrals from orthopedics to general medicine, or on how these referral patterns evolve [8,9]. Insufficient documentation or unclear referral information may compromise continuity of care, potentially leading to delays in clinical decision-making or inappropriate management [10]. Therefore, clarifying the current state of interdepartmental collaboration in community hospitals represents an essential first step toward improving the quality of inpatient care. This study aimed to examine the characteristics and temporal trends in referrals from orthopedics to general medicine at a community hospital, and to explore the underlying challenges and potential strategies to improve collaborative practice. The findings are expected to provide foundational insights to support improvements in interdepartmental collaboration, clinical processes, and educational interventions in community hospital settings.

## Materials and methods

Study design

This study employed an explanatory sequential mixed-methods design. Quantitative data were first collected and analyzed through a retrospective medical record review at Unnan City Hospital, a community hospital located in Shimane Prefecture, Japan, to describe referral patterns and collaboration between the orthopedic and general medicine departments. These findings informed a subsequent exploratory qualitative study using semi-structured interviews with general physicians to identify underlying challenges, perceptions, and potential strategies to improve interdepartmental collaboration.

Setting

The study was conducted at Unnan City Hospital. As of May 2025, Unnan City had a population of 34,055, with an aging rate of 41.19%, substantially higher than the national average [11]. The hospital has 281 inpatient beds and serves a predominantly rural and aging population. A general medicine department was newly established at the hospital in April 2016. The number of full-time physicians in the orthopedic and general medicine departments from fiscal year 2016 to 2024 is shown in Table 1.

**Table 1:** Annual changes in the number of full-time physicians in the orthopedic and general medicine departments

Department	2016	2017	2018	2019	2020	2021	2022	2023	2024
Orthopedics	5	4–5	5	5	5–6	5	3	3	3
General Medicine	1–2	1–2	3	3–4	5	7	8	8	10–11

As of fiscal year 2024, the general medicine department consisted of five to six resident physicians and five staff physicians. During the same period, the internal referral system required the physician on duty for emergency services to simultaneously respond to consultation requests from the orthopedic department.

Participants

Quantitative Component

The quantitative component included all patients aged 18 years or older who were admitted to the orthopedic department at Unnan City Hospital and referred to the general medicine department for any reason during their hospitalization. The study period spanned from April 1, 2016, to March 13, 2024.

Qualitative Component

For the qualitative component, semi-structured interviews were conducted between April 14 and June 6, 2025. All members of the general medicine department who were not involved as co-investigators in the study were invited to participate.

Data collection

Quantitative Data

Data were extracted from the electronic medical records, including patient age, sex, primary diagnosis at admission, length of hospital stay before referral, and the content of referral requests.

Qualitative Data

The first author (KN), a second-year resident in the general medicine department, conducted semi-structured interviews. Each participant completed a one-on-one, face-to-face interview lasting approximately 30 minutes. All interviews were audio-recorded and transcribed verbatim. The interview guide was structured around four core topics: participants’ reflections on the summarized quantitative findings; experiences of difficulty or negative emotions related to referrals; cases in which collaboration with orthopedics was perceived as successful; and perspectives on appropriate referral practices and future collaboration (see Appendices).

Follow-up questions were added flexibly to deepen understanding and explore emerging topics. The interviewer and a co-author (RO), who was an attending general physician, reviewed and discussed the data after each interview. Interview questions were refined iteratively based on emerging insights. Data collection and analysis proceeded concurrently until all eligible participants were interviewed, resulting in a total of 11 interviews.

Data analysis

Quantitative Analysis

Descriptive statistics were used to summarize patient characteristics and referral patterns. Parametric and non-parametric tests were applied to analyze age, sex, and time from admission to referral, as appropriate. Admission diagnoses were categorized and classified, and referral content was grouped into multiple categories through discussion among the authors. Temporal changes in these variables across the study period were described longitudinally.

Qualitative Analysis

The qualitative data were analyzed using thematic analysis following the six-step approach proposed by Braun and Clarke [12]. First, the authors independently read the transcripts to become familiar with the data. Second, transcripts were systematically coded. During open coding, transcripts were segmented into meaningful units and assigned labels and definitions. The first author conducted initial coding, which was subsequently discussed and refined with the co-authors to enhance credibility. During axial coding, relationships among codes were identified, and related codes were grouped into concepts.

In the third step, patterns and relationships among concepts were examined, and codes were collated into candidate themes. In the fourth step, the research team reviewed and refined the themes. In the fifth step, themes were named and clearly defined. In the final step, relationships among themes were identified, and the findings were described narratively. Data collection and analysis were conducted iteratively, and theoretical saturation was confirmed after the 11th interview.

Ethical considerations

This study was approved by the Ethics Committee of Unnan City Hospital (Approval ID: 20240019). All data were anonymized to protect patient confidentiality, and results were reported only in aggregate form. Informed consent for the use of patient information was obtained through a comprehensive institutional consent process.

## Results

Characteristics of the patients referred to general medicine

Over the nine-year study period, 530 patients were identified in electronic medical records as having been referred from the orthopedic department to the general medicine department. The mean age consistently indicated an elderly population, ranging from 81.1 to 86.3 years across the study years. The proportion of male patients varied annually, ranging from 10.7% to 58.3%. The most common reasons for admission each year were orthopedic fractures, particularly vertebral fractures (9-15 cases per year), intertrochanteric femoral fractures (3-20 cases per year), and femoral neck fractures (3-18 cases per year), which together accounted for most admissions. In contrast, pelvic fractures (0-6 cases), spine-related disorders (0-6 cases), pyogenic spondylitis (0-3 cases), and infectious diseases (0-5 cases) were relatively uncommon. Detailed annual patient characteristics and admission diagnoses are summarized in Table 2.

**Table 2:** Patient characteristics and admission diagnoses by year

Item	2016	2017	2018	2019	2020	2021	2022	2023	2024
Number of patients, n	49	71	28	60	54	76	60	75	57
Age (years), mean ± SD	81.1 ± 11.6	84.1 ± 10.4	86.3 ± 9.5	83.4 ± 14.8	84.0 ± 11.3	83.1 ± 11.0	85.4 ± 9.1	85.5 ± 9.5	84.3 ± 11.2
Sex (male), n (%)	14 (28.5)	22 (31.0)	3 (10.7)	23 (38.3)	22 (40.7)	25 (32.9)	35 (58.3)	24 (32.0)	21 (36.8)
Vertebral fracture, n	9	13	10	15	15	13	13	15	12
Intertrochanteric femoral fracture, n	8	14	3	14	9	20	11	13	13
Femoral neck fracture, n	5	8	3	4	8	18	8	11	5
Spine-related disorders, n	4	0	0	2	6	2	2	6	2
Pelvic fracture, n	0	1	2	4	0	1	4	5	6
Multiple trauma, n	0	0	0	2	4	2	2	3	0
Humeral fracture, n	5	1	3	2	2	2	0	5	2
Chronic pain, n	4	7	0	3	1	1	2	2	1
Pyogenic spondylitis, n	0	0	1	1	0	3	1	2	0
Other fractures, n	7	20	3	7	4	9	6	3	10
Other infections, n	1	1	0	0	2	0	3	5	0
Others, n	6	6	3	6	3	5	8	5	6

Referral patterns from orthopedics to general medicine

The annual number of referrals from orthopedics to general medicine ranged from 28 to 76 cases over the nine years. The fewest referrals occurred in 2018 (28 cases), whereas the most happened in 2021 (76 cases). The mean length of hospital stays before referral varied substantially, ranging from 15.8 to 33.1 days, with the shortest interval in 2016 (15.8 days) and the longest in 2019 (33.1 days). The most frequent referral reason across all years was consultation regarding clinical symptoms, accounting for 16-42 cases annually. This was followed by referrals due to laboratory abnormalities (2-17 cases per year) and abnormal vital signs (1-15 cases per year), both of which showed an increasing trend over time. Referrals primarily aimed at comprehensive medical management varied markedly by year, ranging from 0 to 24 cases. In contrast, referrals for imaging interpretation or electrocardiogram evaluation were relatively infrequent. Annual referral volumes, time to referral, and referral reasons are detailed in Table 3.

**Table 3:** Referral volume, time to referral, and reasons for referral by year

Item	2016	2017	2018	2019	2020	2021	2022	2023	2024
Number of referrals, n	49	71	28	60	54	76	60	75	57
Days from admission to referral, mean ± SD	15.8 ± 17.7	14.4 ± 20.6	25.6 ± 28.6	33.1 ± 38.3	21.1 ± 24.8	22.5 ± 25.0	15.9 ± 19.9	24.6 ± 26.7	21.1 ± 24.1
Symptoms, n	33	26	18	32	26	42	31	38	16
Laboratory abnormalities, n	2	9	2	17	15	11	9	12	16
Abnormal vital signs, n	1	8	3	3	6	13	12	15	6
Electrocardiogram, n	0	0	0	3	1	0	0	0	0
Imaging, n	1	1	0	2	2	3	1	0	2
Diagnosis clarification, n	2	3	0	2	1	2	0	4	7
Comprehensive medical management, n	10	24	5	0	3	4	7	6	10
Others, n	0	0	0	1	0	1	0	0	0

Qualitative findings

Thematic analysis identified 13 concepts and four overarching themes. The participants comprised 11 physicians from the orthopedic and general medicine departments (eight men and three women), including six resident physicians and five attending physicians. The four themes developed were: (i) awkwardness in the referral process arising from differences in professional values; (ii) building bridges toward effective collaboration; (iii) mutual collaboration grounded in an appropriate sense of responsibility; and (iv) importance of mutual understanding for smooth collaboration.

These themes indicated that differences in professional values and clinical perspectives between the two departments generated uncertainty and hesitation within the referral process, constituting a fundamental barrier to effective collaboration. In the early stages of establishing collaborative practice, insufficient mutual understanding, rooted in differences in relationships and professional values, emerged as a key challenge. To address these issues, the findings highlighted the need to develop collaborative frameworks grounded in an appropriate sense of shared responsibility, including clarifying roles and refining referral criteria. 

At the same time, participants described psychological and structural barriers to implementing such frameworks, including resistance to increased workload and declining motivation. These barriers were perceived as significant obstacles to sustainable collaboration. Overall, the findings suggest that confronting these challenges directly is a critical first step toward building sustainable referral and collaboration systems in community hospital settings (Table 4).

**Table 4:** Themes and concepts identified through thematic analysis

Theme	Concept
Awkwardness in the Referral Process Arising from Differences in Professional Values	Inadequate communication leading to fragmented collaboration
	Variability in referral practices among orthopedic physicians
	Collaboration failures due to ambiguous responsibility
Building Bridges Toward Effective Collaboration	The need to foster positive interpersonal relationships
	Differences in professional values between departments
	Risks associated with orthopedic-led medical management
Mutual Collaboration Grounded in an Appropriate Sense of Responsibility	Mutual understanding of roles and the importance of flexible transfer of care
	Improving the referral process through system redesign
	Clarification of referral criteria using objective indicators
Importance of Mutual Understanding for Sustainable Collaboration	Shared concerns regarding increasing workload
	Expectations for an expanded role of general medicine
	Challenges related to motivation in general medicine
	The need for sustainable collaborative systems

Theme 1: Awkwardness in the Referral Process Arising from Differences in Professional Values

Inadequate communication leading to fragmented collaboration: Many participants perceived insufficient information sharing between the orthopedic and general medicine departments as a major barrier to effective collaboration. They reported that referral notes documented solely in the electronic medical record often failed to adequately convey the intent or urgency of the referral, making it difficult to respond appropriately.

*“Because the referral note reflected so many mixed intentions, I couldn’t really understand what was being asked, and it led to a poor outcome.”* (Participant A)

Participants also noted that limited opportunities for verbal clarification or informal consultation made it difficult to understand the context or reasoning of the referring physicians, contributing to misunderstandings and emotional friction.

*“When you actually talk, your feelings toward the other person can change. Sometimes negative feelings disappear. You start to understand that there were circumstances behind it.*” (Participant B)

In addition, the infrequency of routine interactions with other departments created psychological barriers to initiating consultations, with some participants describing referrals as “difficult to ask for.” Overall, the lack of mutual understanding and limited dialogue emerged as key factors contributing to delays in care, discrepancies in clinical judgment, and difficulties in building trust between departments.

Variability in referral practices among orthopedic physicians: Participants highlighted considerable inconsistency in referral practices and decision-making criteria among orthopedic physicians. The timing and threshold for referrals were reported to depend heavily on the discretion of the attending orthopedic physician, resulting in situations where patients with similar clinical conditions were referred in some cases but not in others. Attitudes toward the necessity of referral and involvement of other departments were also influenced by individual physicians’ values.

*“Some doctors may want to manage everything themselves, while others may not want too much involvement from another department. Depending on that, our approach to collaboration changes.”* (Participant C)

*“For example, even with fever, some physicians order all the necessary tests before consulting us, while others refer the patient without doing anything. In those cases, it’s hard not to feel negative emotions.”* (Participant D)

Such variability, driven by individual stances and professional values among orthopedic physicians, made it difficult for general medicine physicians to anticipate expectations and plan their responses, thereby undermining the stability of collaboration. These findings suggest that the absence of clear referral criteria and shared interdepartmental guidelines contributes to inconsistencies in care and reduced quality of collaboration.

Collaboration failures due to ambiguous responsibility: Participants emphasized that when orthopedics and general medicine were involved in shared care, the boundaries of roles and responsibilities were often unclear. When accountability was not clearly defined, important clinical decisions could be delayed, and patient management could become fragmented.

*“In shared care, no one really feels like the primary physician, so patients can deteriorate without anyone noticing, and things can go wrong.”* (Participant E)

Some participants expressed the view that assuming consistent responsibility as the primary physician facilitated clearer decision-making and smoother patient care compared with shared care arrangements in which responsibility was diffused.

*“If we’re going to be involved anyway, it might actually be easier to be the primary physician. Then I can decide how aggressively to pursue rehabilitation, whether the patient should go home or to a nursing facility, and take the lead in informed consent discussions, such as those about feeding tubes.”* (Participant F)

These accounts illustrate that ambiguity in responsibility during shared care can lead to delays in decision-making and uneven distribution of workload, ultimately compromising the quality of collaboration. Participants therefore emphasized the need to establish clearer role definitions and accountability structures when implementing shared care models.

Theme 2: Building Bridges Toward Effective Collaboration

The need to foster positive interpersonal relationships: Participants emphasized that establishing positive relationships between the orthopedic and general medicine departments is fundamental to smooth collaboration. In particular, the presence of an approachable atmosphere strongly influenced both the threshold for initiating consultations and the quality of information sharing. Age differences and hierarchical asymmetry were described as factors that made junior physicians hesitant to consult senior orthopedic surgeons, thereby creating barriers to communication.

*“Many of the orthopedic doctors are senior, and they are rarely in the doctors’ office, which makes it difficult to approach them for consultation.” *(Participant B)

Participants also noted that when a sense of mutual support was lacking, clinical workload tended to become unevenly distributed, leading to accumulated dissatisfaction and stress within one department. Conversely, when departments maintained face-to-face relationships and a shared sense of reciprocity, participants felt that a cooperative atmosphere could be sustained even as referral volumes increased.

*“I think being able to see each other’s faces really matters. Even if referrals increase, collaboration feels much smoother when you have a sense that it’s mutual.”* (Participant G)

Several participants further pointed out that a lack of expressed appreciation or respect toward the other department could undermine motivation for collaboration. Collectively, these findings suggest that, beyond improving efficiency through task allocation, sustained and high-quality collaboration depends on mutual respect for professional expertise and a shared commitment to collaboration.

Differences in professional values between departments: Participants described differences in professional values and clinical priorities between orthopedics and general medicine as a fundamental source of difficulty in collaboration. While general medicine emphasizes a holistic perspective encompassing patients’ social backgrounds, activities of daily living, and chronic disease management, orthopedics focuses primarily on acute care and surgical intervention. This divergence was perceived to create clear gaps in clinical perspectives and decision-making criteria.

*“In general medicine, building rapport is important, so being suddenly asked to explain serious issues to families can create negative feelings. Orthopedics often involves surgery on the first day, so I think the nature of the work is quite different.”* (Participant B)

Such differences were also reflected in variations in what was emphasized in medical records, making it more difficult to share a comprehensive understanding of patients. Participants noted that these gaps affected not only treatment strategies but also the pace and urgency of clinical responses, increasing the likelihood of misalignment.

*“There are fundamental discrepancies, like giving intravenous fluids just because a patient isn’t eating. When you think about prognosis, I feel that we might be better positioned to discuss these issues, which makes collaboration challenging.”* (Participant A)

In shared care settings, participants further reported that the intended comprehensive role of general medicine was not always fully understood by orthopedic physicians, limiting opportunities for generalists to contribute their expertise effectively. As a result, there was concern that patient care could proceed without sufficient attention to overall medical management or social context.

Taken together, these findings indicate that differences in professional values between departments extend beyond positional distinctions and influence multiple dimensions of care, including clinical decision-making, information sharing, and response speed, thereby impeding effective collaboration.

Risks associated with orthopedic-led medical management: Participants expressed concern that in community hospitals, many orthopedic inpatients are older adults with multiple comorbidities, yet medical management by orthopedics alone may be insufficient. Because orthopedic care prioritizes acute issues such as fracture management and surgery, chronic disease control and internal medical evaluation were sometimes perceived as being deprioritized.

*“For example, a patient admitted with a femoral neck fracture and multiple comorbidities might continue heart failure medications without reassessment.”* (Participant H)

These accounts suggest inherent limitations in single-department orthopedic management for chronic conditions and acute medical complications, with delays in appropriate interventions potentially leading to clinical deterioration. Consequently, participants emphasized that even for patients admitted primarily for orthopedic conditions, early involvement of general medicine through shared care models is critical for ensuring patient safety and improving the quality of comprehensive medical management.

Theme 3: Mutual Collaboration Grounded in an Appropriate Sense of Responsibility

Mutual understanding of roles and the importance of flexible transfer of care: Participants emphasized the importance of orthopedics and general medicine understanding each other’s professional roles and expertise while flexibly transferring primary responsibility according to patients’ clinical conditions and treatment trajectories. In particular, they noted that appropriately distinguishing between shared care and full transfer of care allowed both departments to maximize their respective strengths.

*“If the patient’s vital signs are stable and they just need antibiotics, but the fracture management is likely to be prolonged, shared care makes sense. On the other hand, if the patient develops sepsis or unstable vital signs, we should switch to a full transfer. I think we need that kind of flexibility.”* (Participant H)

Such flexible decision-making was viewed as dependent on a shared understanding of roles and competencies between departments. Participants also noted that prolonged shared care could lead to ambiguous accountability and delayed responses unless clear criteria were established. In situations involving acute clinical changes, such as abnormal vital signs, early transfer of primary responsibility was considered preferable to maintain continuity and clarity in care.

Conversely, some participants reported cases in which transferring primary responsibility facilitated the development of new trust relationships and led to favorable clinical outcomes. Overall, these findings suggest that mutual trust and context-sensitive adjustment of care responsibilities contribute to safer, higher-quality patient care.

Improving the referral process: Participants indicated that facilitating smoother referrals from orthopedics to general medicine requires reconsideration of both referral workflows and staffing structures. At present, consultation responsibilities are often combined with emergency department duties, creating excessive workload and delays in responding to critically ill patients during periods of high emergency department demand.

*“Having the same person responsible for both emergency care and consultations is a heavy burden. When the emergency department is busy, and a severe consultation comes in, it’s hard to respond adequately.”* (Participant I)

To prevent delays in referral, participants highlighted the importance of early recognition and information sharing by other healthcare professionals, particularly nurses. When nurses identified medical abnormalities early, and orthopedic physicians acknowledged these observations appropriately, earlier involvement of general medicine was considered more likely.

*“Nurses sometimes notice medical abnormalities early, and if orthopedics picks that up, I think it can lead to earlier referrals.”* (Participant E)

At the same time, participants expressed concern that referrals initiated solely by nurses could conflict with orthopedic physicians' intentions. Therefore, they emphasized the need for systems that leverage nurses’ early detection while ensuring that final decisions are shared and coordinated among physicians.

Taken together, these findings suggest that improving referral processes requires redesigning staffing structures with workload considerations in mind and establishing collaborative frameworks that respect multidisciplinary input while clearly defining accountability.

Clarification of referral criteria: Participants identified the lack of clearly defined referral criteria as one factor contributing to delayed consultations from orthopedics to general medicine. Decisions regarding when to seek consultation varied among physicians, leading to inconsistent referral practices.

*“In cases where referrals are delayed, there are often abnormal vital signs that were present earlier. Using vital signs as referral criteria might be helpful, because numbers are easy to understand.”* (Participant E)

Participants suggested establishing referral criteria based on objective indicators, such as vital signs, to standardize decision-making and clarify referral timing. They also noted that explicitly documenting and sharing these criteria would enable not only physicians but also nurses and other healthcare professionals to report patient deterioration more effectively.

*“When there are clear criteria, nurses can say, ‘This patient meets the criteria,’ which makes it easier for them to raise concerns with the orthopedic attending physician.”* (Participant I)

Overall, the establishment of clear referral criteria was viewed as facilitating consultation decisions, fostering shared understanding across the care team, and ultimately improving both the efficiency and quality of interdepartmental collaboration.

Theme 4: Importance of Mutual Understanding for Sustainable Collaboration

Shared concerns regarding increasing workload: Participants expressed concern that as collaboration with the orthopedic department expanded, the workload of the general medicine department increased substantially. An increase in shared care responsibilities and consultation requests made it more difficult to balance inpatient care, outpatient services, and emergency duties, thereby placing strain on the overall clinical system.

“*Unless the orthopedic side is also willing to respond positively to referrals from our department, our workload will just keep increasing, and the collaboration won’t be sustainable.” *(Participant G)

Some participants acknowledged that co-managing all orthopedic patients might improve patient outcomes; however, they also emphasized that such an approach would further increase the burden on general medicine, making long-term, sustainable operations challenging. To maintain ongoing collaboration without compromising the general medicine service, participants stressed the importance of orthopedic physicians understanding the capacity and limitations of the general medicine department and building realistic, mutually supportive collaborative arrangements.

Expectations for an expanded role of general medicine: Participants noted that most orthopedic inpatients in community hospitals are older adults with multiple chronic conditions, and therefore expanding general medicine involvement could lead to improved patient outcomes.

*“Lowering the threshold for consultation makes sense in community hospitals, where many patients are older and orthopedic patients often have multiple comorbidities. If internal medicine were involved in all cases, outcomes might improve.” *(Participant D)

These perspectives suggest that early involvement of general medicine may help prevent inpatient medical complications and support continuity in chronic disease management. Participants also recognized that an expanded role for general medicine would allow orthopedic physicians to focus more fully on surgery and rehabilitation. Overall, active engagement of general medicine was perceived as having the potential to enhance both the quality of orthopedic treatment and comprehensive medical care.

Challenges related to motivation in general medicine: While participants recognized the potential benefits of expanding general medicine involvement, they also described concerns about declining motivation if increased responsibilities were not accompanied by sufficient recognition or structural support. Repeated involvement in complex cases without a clear role definition or acknowledgment was perceived as potentially undermining professional motivation over time.

These findings highlight that although general medicine involvement may improve patient outcomes, sustaining physician motivation requires appropriate role recognition, equitable workload distribution, and organizational support.

The need for sustainable collaborative systems: Participants reported that, at present, the general medicine department has sufficient staffing capacity to accommodate a lower referral threshold. However, they expressed concern that this staffing level might not be maintained in the future, raising doubts about the long-term sustainability of current collaborative practices.

*“Right now, we have enough general medicine physicians to lower the referral threshold, but there’s no guarantee that we’ll always have this many staff, so I worry about whether this approach can be sustained.”* (Participant C)

**Figure 1:**
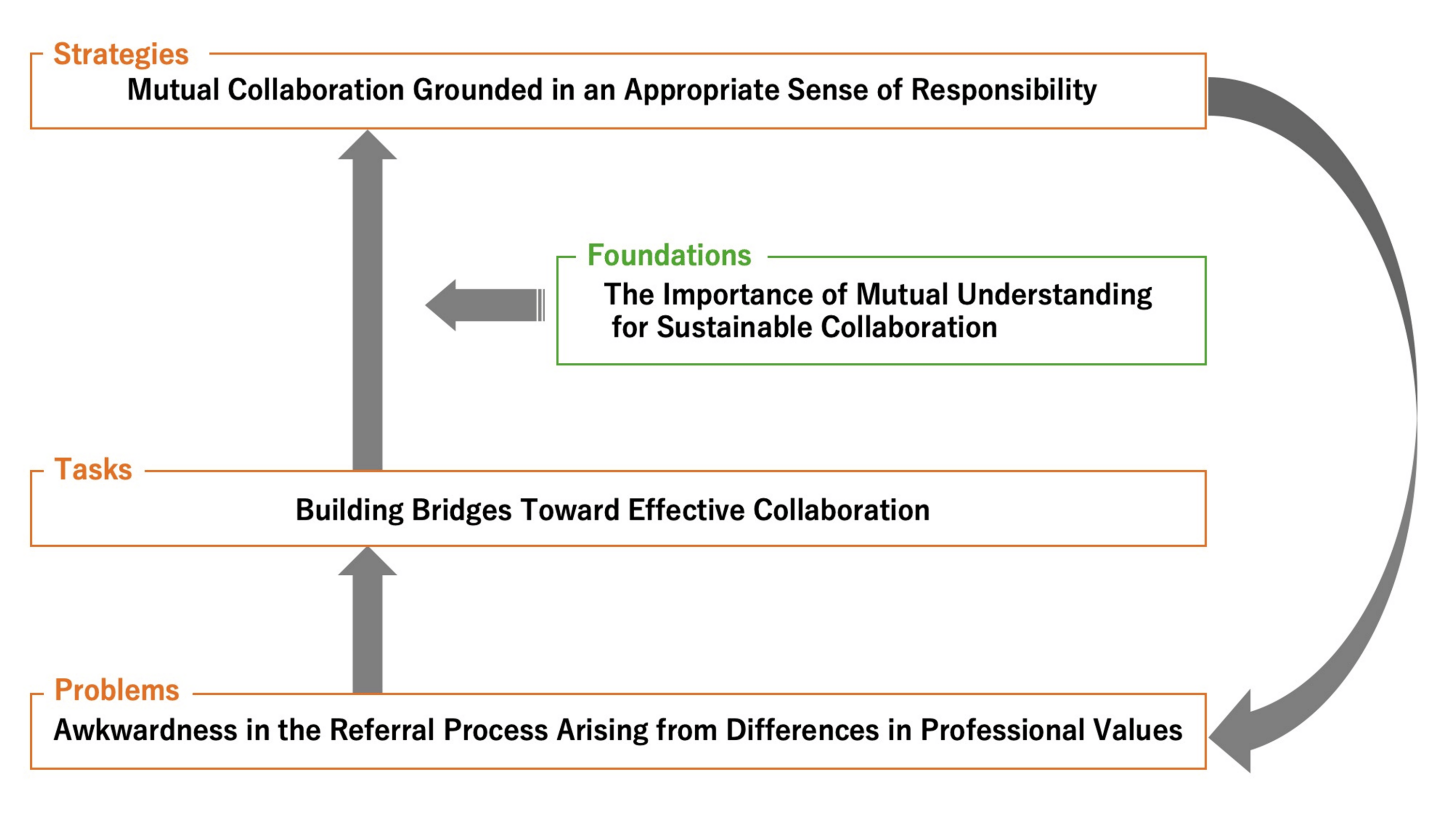
Conceptual model of interdepartmental collaboration between orthopedics and general medicine in a community hospital This figure illustrates the conceptual framework derived from the thematic analysis of interviews with general medicine physicians. Differences in professional values between orthopedics and general medicine contribute to awkwardness in the referral process, including unclear communication, variability in referral practices, and ambiguity in responsibility. These challenges necessitate bridge-building efforts, such as fostering interpersonal relationships and mutual recognition of professional roles. Through these processes, collaboration grounded in an appropriate sense of responsibility can be achieved, supported by flexible transfer of care and clarification of referral criteria. Ultimately, mutual understanding and organizational support are essential for establishing sustainable interdepartmental collaboration in community hospital settings. Image Credit: Authors

These accounts suggest that initiatives based solely on current human resources may be effective in the short term but pose challenges for long-term sustainability. Participants emphasized the need to develop flexible and durable collaborative systems capable of adapting to fluctuations in physician staffing while maintaining quality and safety in patient care (Figure 1).

## Discussion

This study elucidated the impact of establishing a general medicine department in a community hospital, the resulting changes in the management of patients admitted to orthopedic wards, and the challenges associated with further improvement. Differences in professional values and clinical perspectives between orthopedics and general medicine emerged as barriers to collaboration in referral processes, shared care arrangements, and role allocation. At the same time, the findings suggest that the comprehensive perspective provided by general medicine may improve overall medical management and responses to acute clinical deterioration.

In Japan, general medicine physicians and hospitalists are often described as professionals who provide care across a wide range of disease stages, from acute to chronic, while responding to diverse community needs [13]. However, previous studies have noted substantial variation across institutions in the concrete roles and scope of general medicine, with limited standardization [14-16]. The present findings demonstrate how such conceptual-level ambiguity can be reflected in ward-level collaboration and accountability structures.

First, this study revealed that in community hospitals, the role of general medicine in ward management is not always clearly defined, and ambiguity in responsibility during shared care can lead to delays in decision-making and uncertainty in responding to acute clinical changes. These findings are consistent with prior literature on surgical co-management models, which emphasizes that clearly defined accountability and decision-making authority are essential for improving patient outcomes and safety [17,18]. Conversely, early involvement of general medicine, particularly in patients at high risk of clinical deterioration or with unstable general conditions, was perceived as a means of clarifying responsibility and potentially reducing the risk of adverse events [19]. Indeed, multiple studies, including those conducted in Japan, have reported that co-management of orthopedic patients by hospitalists or internists contributes to shorter time to surgery, fewer complications, and reduced length of hospital stay [19,20]. From this perspective, the present qualitative findings provide contextual support for the safety-related contributions that general medicine can make in community hospital settings.

Furthermore, the comprehensive assessment and multimorbidity management competencies inherent to general medicine were perceived as particularly valuable in community hospitals, where a large proportion of inpatients are older adults with multiple chronic conditions. Multimorbidity has been associated with increased readmission risk and poorer inpatient outcomes, and the importance of comprehensive medical management alongside fracture treatment has been highlighted in elderly orthopedic populations [21-23]. As participants in this study noted, the ability to continuously manage medical comorbidities without overlooking internal complications, even in patients admitted primarily for orthopedic conditions, represents a core strength of general medicine. Prior reports from Japanese hospitals that have implemented hospitalist models similarly describe improvements in the quality of care for acutely ill older patients [24,25]. The present study extends this literature by elucidating underlying mechanisms through the lenses of role allocation, value differences, and referral processes.

At the same time, participants indicated that institutional understanding of the role of general medicine was insufficient, and that variability among physicians in referral timing, consultation thresholds, and decisions regarding shared care versus transfer of care contributed to increased workload and fragmented collaboration. Such inconsistency in defining the role of general medicine has been repeatedly discussed in the Japanese literature on generalists and hospitalists, particularly as a barrier to evidence generation and performance evaluation [26,27]. The findings of this study underscore the need for hospitals to explicitly redefine the role of general medicine and to establish shared agreements with orthopedics and other departments regarding task allocation and referral criteria [28]. Deepening organizational understanding in this way may promote more proactive engagement by general medicine and ultimately enhance the overall quality of hospital care [29,30].

Taken together, these findings suggest that in community hospitals, general medicine should not be conceptualized merely as a supplementary service for shared care, but rather as a central function supporting the hospital-wide clinical infrastructure. In recent years, proposals regarding the future roles, education, and evaluation of hospitalists and general medicine physicians in Japan have prompted renewed discussion of their positioning within hospital systems. The present study contributes empirical insights from the specific context of orthopedic wards, highlighting concrete challenges and potential directions for improvement. Making the impact of general medicine on ward-based care more visible and exploring new collaborative approaches, such as standardized co-management models, explicit referral criteria, and structured interprofessional collaboration, may represent important first steps toward building sustainable care systems in community hospitals.

Limitations of the Study and Future Directions

This study has several limitations. First, it was a qualitative study conducted at a single institution, and the experiences of participants may have been influenced by local organizational culture, professional relationships, and regional characteristics. Accordingly, the themes and concepts identified may not be directly transferable to other institutions, and caution is warranted when generalizing the findings. Nevertheless, the context-specific insights presented here contribute to the contextual validity that is central to qualitative research. Second, the analysis focused exclusively on collaboration between orthopedics and general medicine, and perspectives related to other departments or hospital-wide management structures were not fully explored. Future research should incorporate viewpoints from additional specialties and healthcare professionals to provide a more comprehensive understanding of interdepartmental collaboration. Third, as an exploratory qualitative study, this research did not evaluate whether the proposed improvements in collaboration or system redesign would lead to measurable improvements in patient outcomes. To assess the impact of these strategies on patient safety, efficiency, and quality of care, future studies employing multicenter designs, interventional approaches, or mixed-methods methodologies will be necessary. Finally, the researchers’ own clinical backgrounds and professional positions may have influenced the analytic process. Methodological rigor could be further strengthened in future research through investigator triangulation and participation of multidisciplinary analysts.

## Conclusions

This study demonstrates that the introduction of a general medicine department in a community hospital has the potential to add new roles and value to the management of patients in orthopedic wards. Active involvement of general medicine may improve comprehensive medical management, responses to acute clinical deterioration, and interdepartmental collaboration, thereby contributing to improved patient outcomes. At the same time, challenges related to role ambiguity and uneven workload distribution were identified, underscoring the need to redefine the role of general medicine at the organizational level and to establish sustainable collaboration models. Through interprofessional collaboration and institutionalized frameworks, the potential of general medicine in community hospitals can be further developed and leveraged to support safe, comprehensive, and sustainable inpatient care.

## Appendices

Semi-Structured Interview Guide

1. How did you perceive the overall trend and characteristics of referrals from the orthopedic department to the general medicine department shown in the summarized quantitative findings?

2. Have you experienced any difficulties, confusion, or negative emotions related to referrals from orthopedics? Please describe specific situations.

3. Can you describe cases in which collaboration with the orthopedic department was perceived as successful? What factors contributed to successful collaboration?

4. What are your perspectives on appropriate referral practices from orthopedics to general medicine and on future interdepartmental collaboration?
